# Peri-Operative Kinetics of Plasma Mitochondrial DNA Levels during Living Donor Kidney Transplantation

**DOI:** 10.3390/ijms241713579

**Published:** 2023-09-01

**Authors:** Marie Kroneisl, Nora A. Spraakman, Jeroen V. Koomen, Zeinab Hijazi, Femke H. Hoogstra-Berends, Henri G. D. Leuvenink, Michel M. R. F. Struys, Rob H. Henning, Gertrude J. Nieuwenhuijs-Moeke

**Affiliations:** 1 Department of Clinical Pharmacy and Pharmacology, University Medical Center Groningen, University of Groningen, Hanzeplein 1, 9713 GZ Groningen, The Netherlands; 2 Department of Anesthesiology, University Medical Center Groningen, University of Groningen, Hanzeplein 1, 9713 GZ Groningen, The Netherlands; 3 Department of Surgery, University Medical Center Groningen, University of Groningen, Hanzeplein 1, 9713 GZ Groningen, The Netherlands; 4 Department of Basic and Applied Medical Sciences, Ghent University, 9000 Ghent, Belgium

**Keywords:** mtDNA, DAMPs, kidney transplantation

## Abstract

During ischemia and reperfusion injury (IRI), mitochondria may release mitochondrial DNA (mtDNA). mtDNA can serve as a propagator of further injury but in specific settings has anti-inflammatory capacities as well. Therefore, the aim of this study was to study the perioperative dynamics of plasma mtDNA during living donor kidney transplantation (LDKT) and its potential as a marker of graft outcome. Fifty-six donor–recipient couples from the Volatile Anesthetic Protection of Renal Transplants-1 (VAPOR-1) trial were included. Systemic venous, systemic arterial, and renal venous samples were taken at multiple timepoints during and after LDKT. Levels of mtDNA genes changed over time and between vascular compartments. Several donor, recipient, and transplantation-related variables significantly explained the course of mtDNA genes over time. mtDNA genes predicted 1-month and 24-month estimated glomerular filtration rate (eGFR) and acute rejection episodes in the two-year follow-up period. To conclude, mtDNA is released in plasma during the process of LDKT, either from the kidney or from the whole body in response to transplantation. While circulating mtDNA levels positively and negatively predict post-transplantation outcomes, the exact mechanisms and difference between mtDNA genes are not yet understood and need further exploration.

## 1. Introduction

Kidney transplantation evolves in terms of quality and donor types but is also becoming more challenging, due to the use of extended criteria donors (ECD) to overcome the persisting donor shortage [1,2,3]. The current lack of accurate strategies to assess organ quality impedes the identification of viable organs suitable for transplantation and the prediction of graft (dys)function early after transplantation. This potentially contributes to both unnecessary discarding of organs and progression of graft injury [4,5]. To evaluate the quality of donor kidneys, various scoring systems have been utilized, with limited usefulness [6]. Similarly, the current use of creatinine and biomarkers such as kidney injury molecule-1 (KIM-1), interleukin-18 (IL-18), liver-type fatty acid-binding protein (L-FABP), and neutrophil gelatinase-associated lipocalin (NGAL) as indicators of graft function and injury often fail to timely, accurately, and consistently reflect the patency of the graft [7,8,9]. Therefore, there is a critical need for specific and sensitive biomarkers to assess the quality of potential grafts and to monitor post-transplant graft function. One of the main and inevitably harmful processes affecting graft quality is ischemia reperfusion injury (IRI). IRI is a risk factor for delayed graft function (DGF), graft rejection, and chronic graft dysfunction, affecting short- as well as long-term graft and patient outcomes [10,11,12]. Pathophysiologically, IRI is a complex phenomenon involving several molecular pathways and injurious cascades [13]. 

Mitochondria play a central role in IRI, as they are most likely the main source of reactive oxygen species (ROS) production and represent a major effector in the regulation of cell death [14]. Mitochondria contain their own mitochondrial DNA (mtDNA) that encodes for proteins of the synthesis machinery and those involved in oxidative phosphorylation [15]. mtDNA is one of the potential targets of the excessive ROS produced during IRI, as it is about 50-fold more sensitive to oxidative damage than the nuclear genome, presumably due to the lack of histones [16,17]. Upon opening of the mitochondrial permeability transition pores (mPTP) due to extensive ROS formation and Ca^2+^ accumulation, mitochondria may release their (damaged) mtDNA into the cytoplasm and eventually into the vascular compartment, where it can function as damage-associated molecular pattern (DAMP) able to activate the innate immune system [18,19]. However, mtDNA can impact the kidney by initiating a switch to the anti-inflammatory M2 macrophages [20]. Hence, mtDNA can serve as a propagator of injury from the initial site of injury to more distant sites, amplifying cell and tissue injury, which leads to the release of more mtDNA, resulting in a vicious circle of injury, but this can also ensue a more beneficial systemic response for the transplant recipient. 

It is of general interest to study innovative non-invasive strategies to reflect current or predict future graft function, as the current strategies are based on more general markers, are only a temporary reflection, or require invasive methods to assess functioning. Our aim was to study the perioperative dynamics of mtDNA by quantifying graft release and plasma levels of mtDNA during living donor kidney transplantation (LDKT) and the potential of these mtDNA levels to serve as markers of graft outcome. Three mtDNA genes were of interest due to their place and function within the mtDNA loop, namely Displacement-loop (D-loop) as part of the non-coding region, and NADH ubiquinone oxidoreductase subunit 1 (ND1) and NADH ubiquinone oxidoreductase subunit 6 (ND6) as representatives of the coding region of the mitochondrial genome. 

## 2. Results

### 2.1. Baseline Characteristics

Baseline characteristics of donors and recipients are summarized in Table 1. 

### 2.2. Dynamics of mtDNA in Different Vascular Compartments

#### 2.2.1. Systemic Venous mtDNA

**Figure 1:**
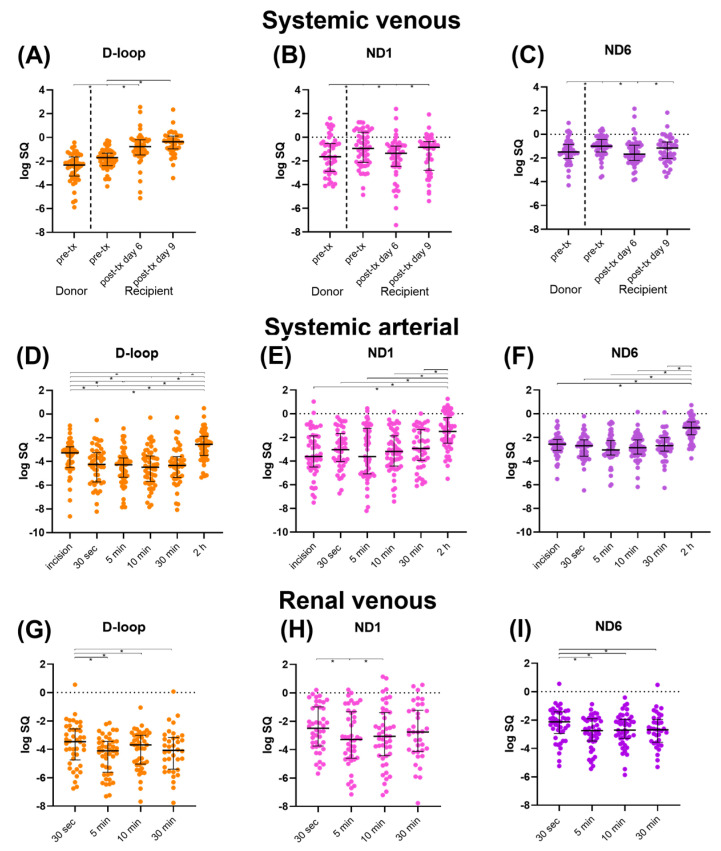
Median mtDNA levels (D-loop, ND1, and ND6) with interquartile range, log transformed. (**A**–**C**): Systemic venous levels. (**D**–**F**): Systemic arterial levels. (**G**–**I**): Renal venous levels. Abbreviations: D-loop: displacement loop, ND1: NADH ubiquinone oxidoreductase subunit 1, ND6: NADH ubiquinone oxidoreductase subunit 6, pre-tx: pre-transplantation, post-tx: post-transplantation. * = *p*-value < 0.05.

Systemic venous mtDNA levels are displayed in Figure 1A–C. Pre-transplantation mtDNA levels of the donor were significantly lower than those of recipients (all *p* < 0.01). In the recipient, D-loop increased between time points (*p* < 0.05), whereas ND1 reached similar levels at day 9 post transplantation compared to pre-transplantation. ND1 significantly increased at day 9 compared to day 6 (*p* = 0.0214). ND6 significantly decreased at day 6 compared to pre-transplantation (*p* < 0.001), followed by an increase at day 9 (*p* = 0.0063). However, ND6 at day 9 was still significantly lower compared to pre-transplantation levels (*p* = 0.0485).

#### 2.2.2. Systemic Arterial mtDNA

Systemic arterial mtDNA levels are displayed in Figure 1D–F. D-loop first significantly decreased compared to the levels after incision (all *p* < 0.05), after which a significant increase at 2 h post-transplantation was seen compared to all previous timepoints (all *p* < 0.001). Systemic arterial ND1 and ND6 levels showed a similar significant increase at 2 h post-transplantation compared to all previous timepoints (all *p* < 0.001). ND6 at 5 min after reperfusion was significantly lower than ND6 after incision (*p* = 0.0344).

#### 2.2.3. Renal Venous mtDNA

Renal venous mtDNA levels are displayed in Figure 1G–I. D-loop levels were significantly higher at 30 s after reperfusion compared to later timepoints (all *p* < 0.05). D-loop at 10 min increased significantly compared to 5 min after reperfusion (*p* = 0.007). ND1 at 5 min was significantly lower compared to 30 s after reperfusion (*p* = 0.0108), followed by a significant increase at 10 min after reperfusion (*p* = 0.0224). ND6 at 30 s after reperfusion was significantly higher compared to later timepoints (all *p* < 0.05).

### 2.3. Plasma mtDNA Variability between Subjects

To study the variance between subjects and the effect of donor, recipient, and transplantation variables on the dynamics of mtDNA, we used linear mixed models (LMM) to analyze the course of plasma mtDNA over time (Table 2). Addition of time and patient ID were tested to develop a crude model for all the different mtDNA genes. 

#### 2.3.1. D-Loop

The slope of the systemic venous model decreased per minute of cold ischemia time (CIT, *p* = 0.0450) but increased (0.0571 logSQ) if the transplantation was pre-emptive (*p* = 0.0492). A female recipient had a higher baseline of the systemic arterial (*p* = 0.0039) and the renal venous D-loop model (*p* = 0.043). 

#### 2.3.2. ND1

The baseline of the systemic venous model was significantly lower, with 0.602 logSQ, if the living donor was unrelated (LURD, *p* = 0.0419). The slope of the systemic venous model significantly increased (0.102 logSQ) if the recipient was female (*p* = 0.0265) and if a sevoflurane-based anesthetic regimen, compared to propofol-based, was applied during transplantation (*p* = 0.0389). The model performed best with the combination of LURD on the intercept and recipient sex on the slope. The slope of the systemic arterial model significantly decreased per minute of CIT (*p* = 0.0436). 

#### 2.3.3. ND6

The second warm ischemia time (WIT2) significantly decreased the slope of the systemic venous model with every minute increase in WIT2 (*p* = 0.0435). The slope of systemic arterial significantly decreased with every minute increase in CIT (*p* = 0.0046). 

### 2.4. Prediction of eGFR Using mtDNA Variables

mtDNA variables were tested in a univariable manner in a linear mixed model for eGFR till two-year follow-up (Table 3). Systemic arterial D-loop 2 h after reperfusion significantly explained the baseline of the eGFR model, thus the 1-month eGFR estimation, with an increase of baseline of 3.34 (SE 1.57) mL/min for every log unit increase of D-loop (*p* = 0.0392). Every log unit increase of systemic venous ND1 at day 9 after transplantation also significantly increased the baseline, with 2.92 (SE 1.23, *p* = 0.022). 

The absolute slope of mtDNA was not a significant predictor for the baseline or slope of the eGFR model. The slope of mtDNA was divided in two distinct categorical groups, namely a declining and increasing group. The increasing ND6 group had a higher baseline of eGFR (10.26 mL/min) compared to the declining group (*p* = 0.0076). 

Significant univariable mtDNA variables were integrated in a multivariable crude model, which was based on significant predictor variables of the donor, recipient, and transplantation (Table 4). Several donor baseline characteristics (age, biological sex, presence of cardiovascular comorbidity, and current smoker) were all significant univariable effects on the baseline of the eGFR model. The data were best described by including donor age in the model (crude model). Addition of the absolute and categorical systemic venous ND1 variables to the baseline improved the model fit and remained significant predictors. Donor age remained significant on the baseline in all models.

### 2.5. Prediction of Acute Rejection Episodes Using mtDNA Variables

mtDNA variables were tested in a univariable manner with a log linear regression model for acute rejection episodes in the two-year follow-up period (Table 3). Systemic venous D-loop levels decreased the odds of acute rejection in the two-year follow-up with every unit (logSQ) increase (*p* = 0.042). Systemic arterial ND1 and ND6 at 5 min after reperfusion increased the odds of acute rejection (*p* = 0.047 and *p* = 0.020, respectively). The slope of ND6, when entered as an absolute value or as a categorical value, decreased the odds significantly (*p* = 0.036 and *p* = 0.022, respectively). Donor, recipient, and transplantation variables were not significant univariable predictors for acute rejection episodes, and we were therefore not able to test the addition of mtDNA predictors to a multivariable model. The predictive quality of mtDNA genes for the occurrence of DGF, graft loss, and patient mortality was not performed due to the low prevalence of these adverse outcomes.

## 3. Discussion

In this post hoc analysis of the VAPOR-1 trial in LDKT, we observed differences in the dynamics of plasma mtDNA genes D-loop, ND1, and ND6, which changed over time and differed between vascular compartments. Additionally, we observed that baseline characteristics were able to explain the variability of mtDNA dynamics between subjects. In addition, both absolute and categorical mtDNA variables positively predicted the baseline of the eGFR model. The prediction for acute rejection episodes with mtDNA variables was ambiguous, and the odds of acute rejection were both higher and lower with absolute and categorical mtDNA variables. 

Our results show that mtDNA can be measured in the systemic venous, systemic arterial, and renal venous compartment of recipients. Donor pre-transplantation mtDNA levels were significantly lower compared to pre-transplantation recipient levels. This is most likely due to the fact that recipients are exposed to long-lasting continuous injurious events as a consequence of their renal disease, dialysis, and other related comorbidities. Eirin and colleagues reinforced this observation by demonstrating that in hypertensive patients urinary mtDNA levels are higher compared to healthy controls and that urinary mtDNA is associated with renal injury markers and graft dysfunction [21]. Post-transplantation systemic venous D-loop levels increased up to day 9 post-transplantation, whereas ND1 and ND6 did not significantly increase compared to pre-transplantation levels. The D-loop gene is part of the non-coding region of mtDNA and perceived as being less vulnerable to (oxidative) stress and injury [22]. A study in patients with a homozygous nonsense mutation in MGME1, encoding a protein needed for mtDNA replication, observed increased levels of D-loop in muscle tissue and fibroblast. The increase of systemic venous D-loop observed by us could be a protective response to the injurious event, by preventing mtDNA rearrangements, as D-loop has important functions for regulation and replication of mtDNA [22,23]. However, the change in systemic venous D-loop was not a significant predictor for kidney function. Another suggestion could be that the regions vulnerable to double-strand breaks and size determines the abundancy of the mtDNA measurements. 

Systemic arterial levels of mtDNA genes showed similar patterns of release, with stable mtDNA levels up to 30 min after reperfusion, followed by a significant increase at 2 h post-transplantation. This time window of the reperfusion period is a known feature of the extensive amount of oxidative stress associated with IRI [13]. ROS and calcium dysregulation are mainly responsible for the opening of the mitochondrial permeability transition pore and thus the release of mtDNA. The delay in the increase in the levels of the mtDNA genes for all three mtDNA genes may indicate that the damage and release process takes considerable time. Alternatively, systemic arterial increase of mtDNA may reflect systemic stress and injury [24,25]. This general stress response and systemic increase in mtDNA was observed in a study focusing on mtDNA levels in ICU patients. Higher ND1 levels were measured in patients who died within 28 days after ICU admission. Patients with sepsis or acute respiratory distress syndrome had significantly higher levels of mtDNA copies compared to patients without these specific diseases [26]. Unfortunately, the origin of the mtDNA genes and its pathological role in initiating, propagating, and limiting diseases remains unknown in these specific clinical settings. 

To our knowledge, this is the first study to have investigated mtDNA release derived directly from the kidney after reperfusion. Renal venous mtDNA levels were highest 30 s after reperfusion. As IRI inevitably occurs in transplantation, and as mitochondrial dysfunction has a central role in IRI, mtDNA is expected to be released from the transplanted kidney into the renal circulation of the recipient. In combination with a more local representation of injury and likely urinary excretion of mtDNA, this might explain the observation of lower concentrations of mtDNA in renal venous samples. It is interesting that renal venous mtDNA, reflecting local renal IRI, occurs in the early reperfusion period, whereas the systemic arterial increase occurs at 2 h post transplantation. This might reflect the capacity of mtDNA to propagate downstream activation of injurious pathways [16,18,24]. However, we had no way to sample plasma from the renal venous compartment at 2 h after reperfusion and are therefore ignorant about these mtDNA levels. Another possibility is the presence of additional efflux routes, such as urinary release or via the lymph system, that might be of greater importance. 

It is important to account for variable patterns of mtDNA levels between patients. Our study showed that a female recipient had an increased baseline of systemic arterial and renal venous D-loop. Several studies have demonstrated that biological sex differences between donor and recipient impacts graft outcome [27,28,29]. In addition, it is known that, in general, women have significantly higher mitochondria-related biomarkers, such as respiration and ATP-content, which has been demonstrated in multiple organs and study models [30,31]. It would be very interesting to study the influence of sex difference on the response to oxidative stress and/or mitochondrial maintenance. We also observed the influence of sex mismatch on biomarkers in a previous study concerning urinary biomarkers [32]. In general, our study demonstrated that various donor, recipient, and transplantation-related factors impacted the baseline or the change of mtDNA over time. Factors that are known to be harmful for transplantation (e.g., longer ischemia times) decreased the baseline or change of mtDNA models. On the other hand, factors known to be beneficial (e.g., pre-emptive transplantation) for transplantation increased the baseline or change of mtDNA models. mtDNA was generally viewed as a damage marker, and therefore our results contradict the current literature. Whether an increase in mtDNA levels at the start of measurement or a positive change of mtDNA over time are truly beneficial remains to be investigated. It may be argued, in combination with the positive predictive functions of mtDNA for eGFR, that release of mtDNA does not solely have a role in injury but might be functional as well, depending on the intensity of the initiating injury and the quality of the subsequent stress response [19]. 

To our knowledge, to date, no study has investigated the predictive qualities of plasma mtDNA in a living donor kidney transplantation cohort. Accumulating evidence has identified plasma mtDNA as a pathogenic amplifier of injury in various clinical conditions, including in Alzheimer’s disease and sepsis [21,25,33,34,35,36,37,38,39,40]. In acute kidney injury (AKI), urinary mtDNA levels were inversely correlated with the eGFR and predicted progression of AKI [37]. In kidney transplantation, mtDNA levels in the urine measured in the early post-transplant period were previously found to be associated with DGF, acute rejection in graft biopsy, and short-term post-transplant renal function [38]. In addition, high plasma levels of mtDNA in deceased donors constitute a risk factor for DGF, suggesting mtDNA as a potential predictive marker of kidney function after deceased donor kidney transplantation [40]. It may be of added value that mtDNA levels measured in the first 9 days after transplantation predict graft outcome, which could lead to patient-centered tailoring of the follow-up, with possibly more or less intensive check-ups and preventive treatment strategies. Current strategies, such as creatine clearance and GFR measurements, only reflect current functioning of the graft and are relatively “late” in showing inferiority. This, thus, does not provide a window of opportunity to intervene in a timely manner. 

Although kidneys derived from deceased donors are exposed to a far greater extent of IRI, kidneys derived from living donors also experience IRI that impacts outcomes. In line with previous studies, our results demonstrated that systemic arterial ND1 and ND6 levels measured after reperfusion positively predicted acute rejection episodes, with higher levels at 5 min after reperfusion increasing the odds of an acute rejection episode. Contrary to these results, we demonstrated that higher mtDNA variables resulted in a higher baseline of the eGFR model. Therefore, it remains unclear to what extent mtDNA levels are predictive of optimal or suboptimal graft outcomes. It is of importance to mention that mtDNA increased the baseline of the eGFR model, which is representative of 1-month eGFR. The outcome of acute rejection episodes in a two-year follow-up period is a categorical variable, for which we did not account for a time factor. Factors of transplantation (e.g., biological sex and ischemia times), but also differences between the function of the three mtDNA genes, may have an influence on the predictive capacity. It might also be the case that a certain threshold of mtDNA is needed for physiological functioning, and beneficial for repair and mitochondrial genome stability, but when exceeded, cellular injury and pro-inflammatory immune activation occurs. As our cohort consisted of high-quality organs, with minimal IRI, this range of mtDNA levels may not have been observed in our study. A phenotypic switch from pro-inflammatory M1 macrophages to anti-inflammatory M2 macrophages could explain this dual role of mtDNA [20]. 

The strengths of our study include the measurement of donor mtDNA levels and the sequential measurements over time in recipients, providing valuable insights into the dynamics of mtDNA. Furthermore, we measured mtDNA in different vascular compartments (systemic venous, renal venous, systemic arterial) collectively covering samples that would be easily collectable in a routine clinical setting. However, similar timepoints of collection may be more beneficial for comparison between vascular compartments. This post hoc analysis of the VAPOR-1 study was limited by a small cohort of 56 patients receiving a high-quality kidney with a low incidence of inferior post-transplantation outcomes, due to limited IRI during LDKT. However, we did find interesting significant predictive functions of mtDNA for graft outcome. An important limitation to acknowledge is that we did not correct for multiple testing. Our study was of explorative design, and we considered that multiple testing correction was not warranted. Furthermore, in this study, mtDNA damage was not assessed to gain knowledge about the quality of mtDNA. In addition, excretion of mtDNA was not studied, and therefore we did not elaborate on the kinetics of mtDNA. 

In conclusion, this study is the first to explore plasma mtDNA in a LDKT cohort and in the renal graft effluent. Our findings indicate that plasma mtDNA has the potential to be of added value for the current screening methods of graft potency. mtDNA variables were predictive for optimal and suboptimal graft outcome parameters, and additional research in a validation cohort is warranted. Additional research is also needed into the function of the different mtDNA genes and the variability between patients. Furthermore, urinary mtDNA also has great potential to be a non-invasive injury marker of kidney injury and repair, and therefore we will proceed by studying urinary mtDNA in recipients of the VAPOR-1 trial. 

## 4. Materials and Methods

### 4.1. Study Design

This study was a post hoc analysis of the Volatile Anesthetic Protection of Renal Transplants-1 (VAPOR-1) trial: a prospective randomized controlled pilot project conducted at the University Medical Centre Groningen (UMCG) between September 2010 and October 2014. Details of this trial were published previously [41]. The Institutional Review Board of the University Medical Centre of Groningen approved the study protocol of VAPOR-1 (METc 2009/334), which was conducted in adherence to the Declaration of Helsinki and registered with ClinicalTrials.gov: NCT01248871. Patients of age ≥18 years, donating the left kidney with a written consent were included. Exclusion criteria comprised of ABO-incompatible transplantation, altruistic donors, and BMI of ≤17 or ≥35 kg.m^−2^. Patients were randomly assigned to one of 3 anesthetic groups: PROP, propofol for donor and recipient; SEVO, sevoflurane for donor and recipient; PROSE, propofol for donor and sevoflurane for recipient. Three couples were excluded from the primary analysis, due to violation of the surgical or immunosuppressive protocol, leaving 57 couples (57 donors and 57 recipients) for analysis. For this analysis, 56 couples, based on available plasma samples, were pooled into one group. 

### 4.2. Sample Size Calculation

Due to the lack of data on circulating mtDNA over time, especially its profile and variation in renal transplant patients, we were unable to perform an exact sample size calculation. Therefore, we calculated the number of predictors that could be assessed assuming a model with *p* < 0.05, while preserving an adequate beta (>0.8). For small, medium, and large effects (f2 =0.02, 0.15, and 0.35, respectively), the maximal number of predictors was not determined, 2, and 10, respectively. Based upon this calculation, we concluded that the number of samples/patients included in the VAPOR1 trial was sufficient for this study to detect medium and large differences in plasma mtDNA levels between the various groups and time points.

### 4.3. Outcome Measures

**Figure 2:**
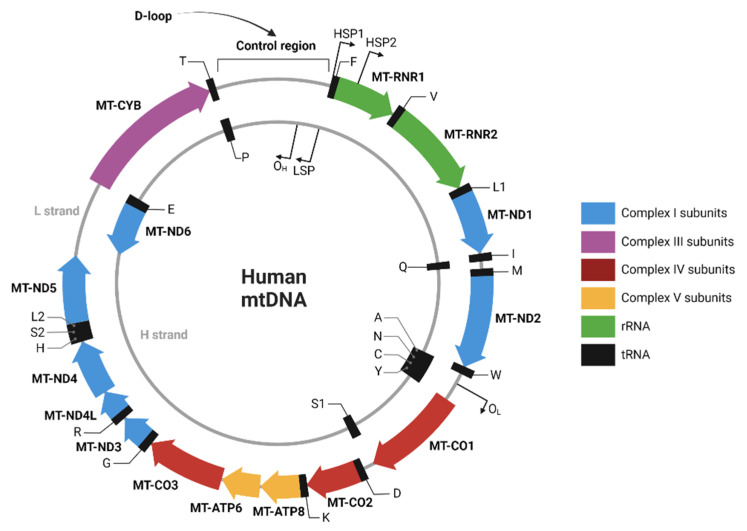
Human mtDNA sequence map. Created with BioRender.com (created and accessed on 4 November 2022).

The primary outcome of this study was to measure 3 different mtDNA genes: D-loop, ND1, and ND6 at several peri-operative timepoints and in several vascular compartments (systemic venous, systemic arterial, and renal venous). D-loop was chosen as it is part of the non-coding region of mtDNA and therefore assumed to be less prone to damage compared to the ND1 and ND6 genes. ND1 and ND6 were chosen as they encode proteins involved in the respiratory chain of the mitochondria but differ in localization with respect to the L- and H-strand (Figure 2). The difference between the clinical values of the different subunits is still unknown and based on assumptions. Therefore, we aimed to cover all areas of interest concerning the different of mtDNA regions. Secondary outcomes were the relation of mtDNA levels to glomerular filtration rate (GFR), occurrence of DGF (defined as need for dialysis in the first week after transplantation), acute rejection episodes (defined as treated and biopsy-proven), graft loss, and patient mortality. Pre-donation kidney function in donors was assessed with mGFR, with use of iodine 125-iothalamate, performed at least 3 months before donation. In recipients eGFR was calculated with the use of the CKD-EPI formula at month 1, 3, 6, 12, and 24 months post transplantation.

### 4.4. Timepoints

**Figure 3:**
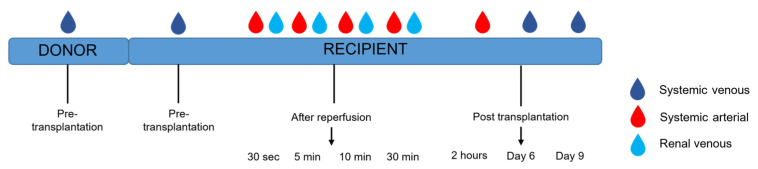
Timeline sample collection in donor and recipient.

Multiple vascular compartments were sampled, as mtDNA can potentially function differently in different vascular compartments. In addition, the dynamics of mtDNA in a vascular compartment and shifts between compartments were of interest for our study. Systemic venous mtDNA levels were measured in plasma obtained from the donor at the pre-transplantation timepoint. Systemic venous samples were collected from the recipients at various time points: pre-transplantation and post-transplantation (day 6 and day 9). Systemic arterial samples were collected after incision, after reperfusion (30 s, 5 min, 10 min, and 30 min), and post-transplantation (2 h). After reperfusion, renal venous were taken from the renal vein with the use of a gonadal vein catheter (30 s, 5 min, 10 min, and 30 min). Timepoints are depicted in Figure 3.

### 4.5. mtDNA Analysis

Isolation of mtDNA from plasma was performed with a Maxwel^®^ RSC ccfDNA Plasma Kit (Promega, Madison, WI, USA), according to a protocol using 50 µL of plasma and 50 µL of elution buffer. Subsequently, the isolated plasma mtDNA was quantified with a CFX384- Real-Time system (Biorad, Hercules, CA, USA) targeting human mitochondrial D-loop, NADH ubiquinone oxidoreductase subunit 1 (ND1), and NADH ubiquinone oxidoreductase subunit 6 (ND6) genes. Clone manager 9 software was used to design the selected primers (Sigma Aldrich, Darmstadt, Germany), which were subsequently validated by assessing the efficiency, melting, and temperature curves. All reactions were carried out in duplicate, and the obtained threshold cycles (ct) values were averaged. A standard curve was generated using a determined concentration of human endothelial kidney cells 293. 

### 4.6. Statistical Analysis

Statistical analysis was performed in SPSS version 23 (IBM Corp, Armonk, NY, USA), R (version 4.2.2) with R Studio (version 2022.12.0.353, PBC, Boston, MA, USA) and GraphPad Prism version 8.0.1. (GraphPad software, Inc, La Jolla, CA, USA). Continuous variables were tested for normal distribution with use of the Shapiro–Wilk normality test and visualized using normal probability (Q-Q) plots. Descriptive statistics were presented as mean (±SD) for normally distributed variables, median (interquartile range (IQR)) in case of non-normal distributions, or proportions *n* with corresponding percentages (%) for categorical variables. mtDNA data were log-transformed. Depending on the normality distribution, independent variables were tested using a *t*-test or Mann–Whitney U test. For dependent variables, a paired *t*-test or Wilcoxon signed rank test was performed. Both mtDNA levels as single timepoints and as the slope (categorical and absolute) of mtDNA change over time were used for prediction analyses. mtDNA levels that were predictive for outcomes in a univariable manner were tested in a multivariable model, to analyze the additional value of mtDNA in prediction models. Linear mixed models were used to assess changes in mtDNA and eGFR over time, where time was entered as a fixed effect. As time was entered as a covariable, we tested polynomials to see which model best described the change over time. Estimates of covariables are presented as b with standard error (SE). Effects for patients, baseline characteristics (based on literature), and time were tested to improve the model. Best-fitted models are presented based on the log-likelihood statistics and the information criteria. For acute rejection episodes as an outcome, a logistic regression was performed, and data were depicted as a odds ratio (Exp(B) with 95% confidence interval). Statistical significance was set at a *p*-value ≤0.05 for all comparisons.

## Figures and Tables

**Table 1:** Baseline characteristics of donors and recipients.

Donor	*n* = 56
Age (y)	52.5 (±10.9)
Male (*n* (%))	26 (46.4%)
BMI (kg/m^2^)	27 (±3.2)
Active smokers (*n* (%))	16 (28.6%)
Cardiovascular comorbidity (*n* (%))	17 (30.4%)
Medication use (*n* (%))	
*Antihypertensive therapy*	15 (26.8%)
*Statins*	7 (12.5%)
*PPI’s*	9 (16.1%)
Pre-donation mGFR (mL/min)	111 (97–135)
Recipient	*n* = 56
Age (y)	53.5 (45.3–58.8)
Male (*n* (%))	26 (46.4%)
BMI (kg/m^2^)	24.8 (22.4–27.7)
Cardiovascular comorbidity (*n* (%))	38 (67.9%)
Medication use (*n* (%))	
*Antihypertensive therapy*	51 (91.1%)
*Phosphate binders*	31 (55.4%)
*Statins*	27 (48.2%)
Unrelated donor (*n* (%))	28 (50%)
Pre-emptive transplantation (*n* (%))	27 (48.2%)
Re-transplantation (*n* (%))	7 (12.5%)
≥3 HLA mismatches (*n* (%))	34 (60.7%)
Positive PRA (*n* (%))	7 (12.5%)
Ischemia times (min)	
*WIT 1*	4 (3–4)
*CIT*	172.5 (155.5–187.5)
*WIT 2*	42.9 (±7.4)
Kidney and patient outcomes	*n* = 56
DGF (*n* (%))	3 (5.4%)
eGFR 1 month post transplantation (mL/min/1.73 m^2^)	51.0 (±14.9)
eGFR 3 months post transplantation (mL/min/1.73 m^2^)	46.2 (38.8–58.6)
eGFR 6 months post transplantation (mL/min/1.73 m^2^)	47.6 (38.6–61.1)
eGFR 12 month post transplantation (mL/min/1.73 m^2^)	50.2 (±14.4)
eGFR 24 month post transplantation (mL/min/1.73 m^2^)	51.8 (±17.6)
Acute rejection 2 years (*n* (%))	9 (16.1%)
Graft loss (*n* (%))	2 (3.6%)
Mortality (*n* (%))	1 (1.8%)

Data given as mean (±SD), median (IQR), or *n* (%). Abbreviations: BMI: body mass index; PPI’s: proton pump inhibitors; GFR: glomerular filtration rate; HLA: human leukocytes antigens; PRA: panel specific antibodies ≥ 15%; WIT1: warm ischemia time 1; CIT: cold ischemia time; WIT2: warm ischemia time 2, DGF: delayed graft function, mGFR: measured glomerular filtration rate, eGFR: estimated glomerular filtration rate.

**Table 2:** Univariable mixed models of plasma mtDNA.

	Systemic Venous			Systemic Arterial			Renal Venous		
Variables	B	SE	*p*	B	SE	*p*	B	SE	*p*
**D-loop**									
Recipient sex (I) Male = 0, female =1				0.95	0.32	0.0039	0.79	0.38	0.043
Pre-emptive tx (S) No = 0, yes = 1	0.057	0.029	0.0492						
CIT (S) In minutes	−0.001	0.00050	0.0450						
**ND1**									
Unrelated donor (I) No = 0, yes = 1	−0.60	0.29	0.0419						
Recipient sex (S) Male = 0, female =1	0.10	0.045	0.0265						
Anesthetic regimen (S) * SEVO vs. PROP	0.12	0.057	0.0389						
CIT (S) In minutes				−0.000098	0.000045	0.0313			
**ND6**									
WIT2 (S) In minutes	−0.0041	0.002	0.0435						
CIT (S) In minutes				−0.000088	0.000031	0.0046			

Linear mixed models were used to study the dynamics of plasma mtDNA. Variables were tested on the intercept (I) and the slope (S) of the mixed model. Best-fitted models are presented with estimates of fixed effects (b with SE). Statistical significance was set at *p*-value < 0.05. * Significant different between anesthetic regimen with SEVO for donor and recipient, having a significantly higher baseline compared to PROP for donor and recipient. Abbreviations: D-loop: displacement loop, ND1: NADH ubiquinone oxidoreductase subunit 1, ND6: NADH ubiquinone oxidoreductase subunit 6, I: intercept, S: slope, B: estimate, SE: standard error, tx: transplantation, WIT2: warm ischemia time 2, BMI: body mass index, LURD: living unrelated donor, GFR: glomerular filtration rate; CIT: cold ischemia time.

**Table 3:** Univariable testing for the prediction of eGFR and acute rejection episode.

eGFR			Acute Rejections Episodes		
**Single Timepoints of mtDNA**					
Variable	*p*-value	Estimate with SE	Variable	*p*-value	Exp(B) with 95% CI
SA D-loop (logSQ) 2 h after reperfusion	0.0392	3.34 (1.57)	SV D-loop (logSQ) Donor pre-transplantation	0.042	0.52 (0.28–0.98)
SV ND1 (logSQ) Day 9 post transplantation	0.022	2.92 (1.23)	SA ND1 (logSQ) 5 min after reperfusion	0.047	1539 (1.01–2.36)
			SA ND6 (logSQ) 5 min after reperfusion	0.020	2.61 (1.16–5.84)
**Slopes of mtDNA (categorical)**					
Variable	*p*-value	Estimate with SE	Variable	*p*-value	Exp(B) with 95% CI
SV ND6 Negative slope = 0 Positive slope =1	0.0076	10.26 (3.69)	SA ND6 Negative slope = 0 Positive slope =1	0.036	0.14 (0.02–0.88)
**Slopes of mtDNA (absolute)**					
Variable	*p*-value	Estimate with SE	Variable	*p*-value	Exp(B) with 95% CI
			SA ND6 (logSQ)	0.022	0.30 (0.11–0.84)

Linear mixed models were used to study the dynamics of eGFR. mtDNA variables were tested on the intercept (I) and the slope (S) of the mixed model. Best-fitted models are presented with estimates of fixed effects (b with SE). Statistical significance was set at *p*-value < 0.05. For acute rejection, a logistic regression analysis was performed, and results with an estimate (Exp (B) with 95% CI). Abbreviations: eGFR: estimated glomerular filtration rate; SE: standard error; CI: confidence interval; SV: systemic venous; SA: systemic arterial; D-loop: displacement loop; ND1: NADH ubiquinone oxidoreductase subunit 1, ND6: NADH ubiquinone oxidoreductase subunit 6.

**Table 4:** Multivariable testing for the prediction of eGFR.

eGFR			
	Variable	*p*-Value	Estimate with SE
Crude model	Donor age (y)	<0.001	−0.66 (0.14)
Addition of single time-points mtDNA	SA D-loop (logSQ) 2 h after reperfusion	0.0817	2.46 (1.38)
	SV ND1 (logSQ) Day 9 post transplantation	0.0131	2.63 (1.02)
Addition of slope of mtDNA	SV ND6 Negative slope = 0 Positive slope =1	0.0204	7.81 (3.26)

Linear mixed models were used to study the dynamics of eGFR in a multivariable manner. Donor, recipient, and transplantation-related variables were tested in a univariable manner, from which a crude model was built. mtDNA variables were tested on the intercept (I) and the slope (S) of the crude model. Best-fitted models are presented with estimates of fixed effects (b with SE). Statistical significance was set at *p*-value < 0.05. Abbreviations: eGFR: estimated glomerular filtration rate; SE: standard error; SV: systemic venous; SA: systemic arterial; D-loop: displacement loop; ND1: NADH ubiquinone oxidoreductase subunit 1, ND6: NADH ubiquinone oxidoreductase subunit 6.

## Data Availability

The data that support the findings of this study are available from the corresponding author upon reasonable request.
